# A positive association of serum CCN5/WISP2 levels with the risk of developing gestational diabetes mellitus: a case–control study

**DOI:** 10.1186/s12576-023-00879-z

**Published:** 2023-10-04

**Authors:** Mohammed Farhan Hamdan Alshganbee, Fariba Nabatchian, Vida Farrokhi, Reza Fadaei, Nariman Moradi, Reza Afrisham

**Affiliations:** 1 Department of Clinical Laboratory Sciences, School of Allied Medical Sciences, Tehran University of Medical Sciences, Tehran, Iran; 2 Department of Hematology, Faculty of Allied Medical Sciences, Tehran University of Medical Sciences, Tehran, Iran; 3 Sleep Disorders Research Center, Kermanshah University of Medical Sciences, Kermanshah, Iran; 4 Liver and Digestive Research Center, Research Institute for Health Development, Kurdistan University of Medical Sciences, Sanandaj, Iran

**Keywords:** CCN5/WISP2, Gestational diabetes mellitus, Inflammation, Pregnancy, Women

## Abstract

**Introduction:**

CCN5/WISP2 is prominently manifest in adipose tissue and has been linked to the pathogenesis of obesity, diabetes, and insulin resistance. However, discrepancies exist in previous studies, and little is known about its association with gestational diabetes mellitus (GDM). The current investigation is designed to examine the correlation of WISP2 with risk factors in GDM patients in comparison to healthy pregnant women for the first time.

**Methods:**

This case–control study measured serum levels of CCN5, TNF-α, IL-6, adiponectin, and fasting insulin using ELISA kits in 88 GDM patients and 88 pregnant women.

**Results:**

The GDM group had remarkably higher serum levels of CCN5 (379.41 ± 83.078 ng/ml) compared to controls (212.02 ± 77.935 ng/ml). In a similar vein, it was observed that patients diagnosed with GDM exhibited elevated levels of pro-inflammatory cytokines such as IL-6 and TNF-α; while conversely, adiponectin levels were found to be significantly lower than those observed in the control group (*P* < 0.0001). In women with GDM, a positive and significant correlation was observed between CCN5 and BMI, FBG, insulin, HOMA-IR, as well as IL-6 and TNF-α levels. In the adjusted model, the risk of GDM was significantly increased with elevated serum CCN5 level.

**Conclusion:**

Our research indicates a noteworthy and affirmative correlation between the levels of CCN5 in the serum and the risk of developing GDM, along with its associated risk factors such as BMI, insulin resistance index, FBG, and inflammatory cytokines (TNF-α and IL-6). These findings suggest that CCN5 could potentially play a role in the etiology of GDM.

## Introduction

Gestational diabetes mellitus (GDM) stands out as a highly prevalent complication encountered during the course of pregnancy [1]. The chief etiological factors responsible for GDM are glucose intolerance and insulin resistance [2]. During the second trimester, insulin resistance is accentuated, which results in elevated blood glucose levels that support fetal growth. However, if there is an insulin resistance or malfunction in beta-cell function remains unchecked, GDM manifests [3]. Furthermore, the likelihood of type 2 diabetes (T2DM), preeclampsia, stillbirth, and cardiovascular diseases is significantly elevated in women with GDM. Offspring born to mothers diagnosed with GDM are susceptible to developing jaundice, polycythemia, hypocalcemia, respiratory distress syndrome, and hypoglycemia are more likely to suffer from diabetes and obesity in their adult years [4–6]. Notably, changes in the levels of steroid hormones (e.g., cortisol, progesterone, estrogen) and placental hormones (e.g., placental lactogen hormone) can exacerbate insulin resistance [3, 7].

Obesity represents a significant risk factor for GDM [8], with evidence from previous studies indicating lipid accumulation in the muscle and liver of GDM patients [9, 10]. It is widely acknowledged within academic circles that a significant association exists between obesity, inflammation, and insulin resistance during the gestational period [8, 11]. Dysfunctional adipocytes resulting from obesity disrupt the equilibrium of metabolic mediators, including adiponectin, visfatin, chemerin, resistin, leptin, and inflammatory cytokines, such as tumor necrosis factor alpha (TNF-α) and interleukin-6 (IL-6). These imbalances can result in insulin resistance and exacerbate GDM progression [12–14]. Furthermore, some of these mediators have demonstrated the ability to impact beta-cell function [15].

An expanding body of evidence suggests that CCN family proteins, a subclass of adipokines, play a vital role in obesity and its associated pathologies [16]. Among these proteins, CCN5, also known as WNT1-inducible-signaling pathway protein 2 (WISP2), exhibits expression in a diverse range of tissues, including adipose tissue, the reproductive tract, the proximal gastrointestinal tract, pancreatic beta cells, and mesenchymal cells [17, 18]. Compelling research reveals that WISP2 expression increases in obesity and insulin resistance [19, 20]. Hammarstedt et al*.* [19] evinced a constructive association linking the expression of WISP2 and the accrual of ectopic adipose tissue, while simultaneously observing an inverse correlation with insulin sensitivity. Their study concluded that WISP2 gene expression was linked to insulin resistance and hypertrophic obesity. In contrast, certain investigations indicate that a deficiency in WISP2 gene expression leads to increased adipogenesis, adipocyte hypertrophy, and fat accumulation [21]. Moreover, Kim et al*.* [21] highlighted that mice lacking the CCN5 gene in their hearts experienced elevated blood glucose levels, ultimately leading to insulin resistance, obesity, and mild diabetes, along with increased pro-inflammatory gene expression. The study's findings showed that increasing WISP2 expression could lead to improved insulin sensitivity and lowered blood glucose levels. Moreover, Alami et al. [22] reviewed that elevated circulating WISP2 levels could improve metabolic status by enhancing the proliferation of pancreatic beta cells.

To date, no investigation has examined the potential link between WISP2 serum levels and gestational diabetes mellitus (GDM) risk in women with GDM in comparison to pregnant females. Given the established connection of CCN5/WISP2 with obesity, diabetes, and insulin resistance, alongside the inconsistencies in earlier research, the present study aimed to explore the association of WISP2 with risk factors in GDM patients. This study seeks to provide a comprehensive understanding of the intricate relationship between CCN5 and metabolic disorders.

## Methods

### Participants

In this study utilizing a case–control design, a group comprising 88 pregnant women in a state of good health was chosen as the control group, while 88 pregnant women with GDM, in accordance with the American Diabetes Association criteria, were included as the case group. The ethics committee of Tehran University of Medical Sciences provided approval for the study (Approval code: IR.TUMS.SPH.REC.1401.264), and all individuals involved in the study were required to provide informed consent by means of affixing their signature to the consent form. The study procedures adhered to the tenets of the Declaration of Helsinki. To be eligible for inclusion, individuals could not have a history of inflammatory diseases, cancer, kidney and liver diseases, thyroid gland disorders, previous diabetes or family history of diabetes, metabolic syndrome, high blood pressure, or be using medical drugs or steroids. The control and GDM cohorts were homogenous in terms of age, body mass index (BMI) and gestational age.

### Physical assessments

The participants' body mass index (BMI) was determined using a standard scale and wall-mounted stadiometer to measure their weight and height, respectively, based on the standard formula [weight (kg)/height (m^2^)]. Furthermore, diastolic and systolic blood pressure at rest were determined using a standard sphygmomanometer.

### Measurement of biochemical parameters

To extract serum, following a period of fasting lasting around 12 h, a venous blood sample of 6 ml was obtained and was centrifuged at 3000*g* for 5 min. The serum levels of fasting blood glucose (FBG) were measured using an autoanalyzer instrument with a kit following the manufacturer's instructions (Pars Azmoun commercial kits, Iran). The insulin levels were ascertained through employment of an ELISA reader tool with a kit in accordance with the manufacturer's guidelines (Monobind; USA). The calculation of the insulin resistance homeostasis model value (HOMA-IR) was performed using the formula [FBG (mg/dl) × [insulin (μU/mL)/405]). To determine the lipid profile, including low-density lipoprotein-cholesterol (LDL-C), triglycerides (TG), high-density lipoprotein-cholesterol (HDL-C), and total cholesterol (TC), an automated analyzer and kits were utilized, as per the instructions provided by the manufacturer (Pars Azmoun commercial kits, Iran) [23].

### Measurement of inflammatory cytokines and adipokines

The ELISA method was utilized to quantify the serum levels of WISP2 and adiponectin, employing kits from MyBioSource and adipogen, respectively. The inter-assay and intra-assay variation coefficients (CV) for WISP2 were < 10% and < 8%, while those for adiponectin were 4.3% and 3.4%, respectively. Additionally, the levels of TNF-α and IL-6 cytokines were determined using an ELISA kit from R&D Systems, following the manufacturer's instructions. The lower limits of detection for WISP2, adiponectin, TNF-α, and IL-6 were 156, 100, 5.5, and 0.11 reported in picograms per milliliter, respectively.

### Statistical analysis

The data underwent statistical analysis utilizing IBM SPSS version 27 (Chicago, USA) and GraphPad Prism 9.5.1.733 software was used to generate the graphs. A *P*-value less than 0.05 was considered as statistically significant. The chi-square test was employed to assess the association between nominal variables. The assessment of data normality was conducted through the utilization of the Kolmogorov–Smirnov test. Subsequently, the Student t-test was applied to data exhibiting normal distribution, and the resultant findings were presented as mean values accompanied by their respective standard deviations (SD). For non-normally distributed data, the U Mann–Whitney test was executed, and the outcomes were reported as median values supplemented by their respective interquartile ranges (IQR). Furthermore, to eliminate the influence of plausible confounding variables on the serum levels of WISP2, an analysis of covariance (ANCOVA) was conducted. The utilization of Pearson correlation analysis was employed to assess the correlation between WISP2 and continuous variables, whereas binary logistic regression was applied to examine the association between WISP2 and the likelihood of GDM. Furthermore, the sensitivity and specificity of WISP2 for diagnosing GDM patients were assessed using the receiver operating characteristic (ROC) curve.

## Results

### Basic anthropometric and biochemical characteristics

Table 1 presents a comparison of the basic anthropometric, immunological, and biochemical characteristics between 88 control individuals and 88 pregnant women diagnosed with GDM. The age and BMI of both the GDM and control cohorts exhibited no significant disparity (*P* = 0.711 and *P* = 0.791, correspondingly). Nevertheless, the tabulated laboratory findings in Table 1 indicate a remarkable variance between the two groups, barring HDL-C (*P* = 0.991). Specifically, the serum levels of insulin, FBG, HOMA-IR, TG, LDL-C and TC were significantly higher in the GDM group than in the control group (*P* < 0.0001).

**Table 1:** Basic anthropometric, immunological, and biochemical characteristics of 88 control people and 88 pregnant women with GDM

Variables	Control		GDM		*P*-value
	Mean ± SD		Mean ± SD		
Age	31.38	4.297	31.60	3.819	0.711
BMI (kg/m2)	27.0795	3.53702	26.9210	4.33222	0.791
SBP (mmHg)	126.61	17.638	133.75	20.214	0.014
DBP (mmHg)	75.25	11.506	79.68	14.791	0.028
FBG (mg/dl)	89.38	12.594	157.98	24.844	< 0.0001
Insulin (µU/ml)	5.089	3.6028	10.568	4.6964	< 0.0001
HOMA-IR	1.1379	0.83638	4.1876	2.14651	< 0.0001
TG (mg/dl)	136.10	51.359	172.99	56.909	< 0.0001
TC (mg/dl)	180.99	44.002	208.72	47.041	< 0.0001
LDL-C (mg/dl)	107.72	34.033	129.20	38.188	< 0.0001
HDL-C (mg/dl)	49.20	9.245	49.19	5.907	0.991
Adiponectin (µg/ml)	14.151	8.0867	9.770	3.6454	< 0.0001
TNF-alpha (pg/ml)	14.470	6.3028	24.669	10.4177	< 0.0001
IL-6 (pg/ml)	5.846	3.2032	9.685	3.5758	< 0.0001
CCN5 (ng/ml)	212.02	77.935	379.41	83.078	< 0.0001

### Levels of cytokines and adipokines

**Fig. 1:**
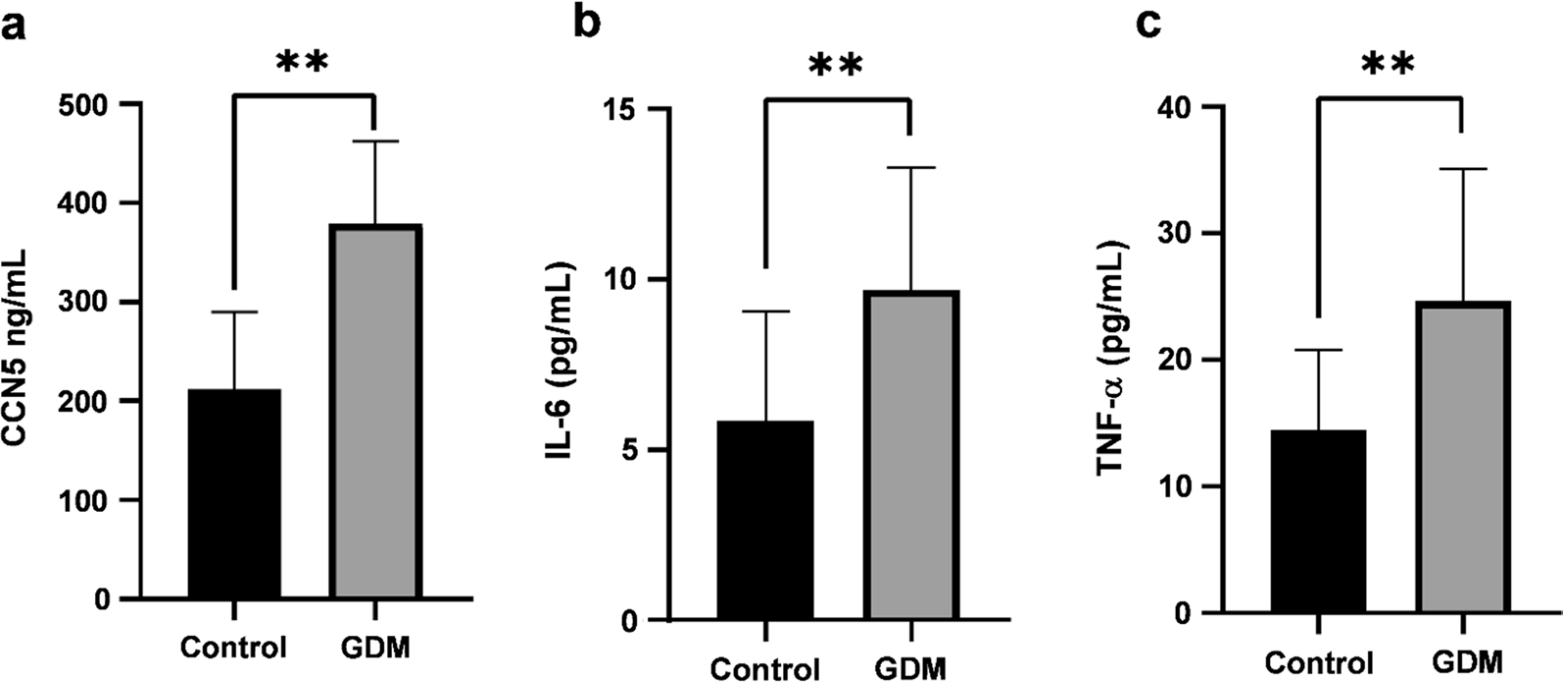
Serum levels of (**a**) CCN5, (**b**) IL-6 and (**c**) TNF-α in the control and GDM groups. All three biomarkers were higher in the GDM group than the control women

The study revealed a marked reduction in adiponectin concentrations among the group with GDM at 9.770 ± 3.654 µg/ml, relative to the control group (14.151 ± 8.086 µg/ml, *P* < 0.0001). Additionally, the serum levels of TNF-α (Fig. 1c and Table 1) and IL-6 (Fig. 1b and Table 1) were significantly higher in the GDM group (24.669 ± 10.417 pg/ml and 9.685 ± 3.575 pg/ml, respectively) than in the control group (14.470 ± 6.302 pg/ml and 5.846 ± 3.203  pg/ml, *P* < 0.0001 for both comparisons, respectively). Furthermore, there was a notable increase in the serum levels of CCN5 in the GDM group (379.41 ± 83.078 ng/mL) compared to the control group (212.02 ± 77.935 ng/mL, *P* < 0.0001) (Fig. 1a and Table 1).

### The correlation of CCN5 level with other variables

To examine the association between CCN5 and other factors, Pearson correlation analysis was conducted, and the results are presented in Tables 2, 3. In the control group, a significant and positive correlation was observed between CCN5 levels and both insulin (*r* = 0.366) and HOMA-IR (*r* = 0.381) levels (*P* < 0.0001 for both). Additionally, there was a significant correlation with adiponectin (*r* = 0.231, *P* = 0.031) and IL-6 (*r* = 0.268, *P* = 0.012) levels. In women diagnosed with gestational diabetes mellitus, a significant and positive correlation was found between CCN5 serum levels and BMI (*r* = 0.251, *P* = 0.019), FBS (*r* = 0.273, *P* = 0.010), insulin (*r* = 0.369, *P* < 0.0001), HOMA-IR (*r* = 0.424, *P* < 0.0001), as well as IL-6 and TNF-α levels (*r* = 0.340, *P* = 0.001 and *r* = 0.253, *P* = 0.018, respectively).

**Table 2:** The correlation analysis of various variables with CCN5 in the control group

Variables	*r*	*P*-value
Age	− 0.179	0.096
BMI	0.140	0.194
SBP	− 0.188	0.080
DBP	− 0.140	0.194
FBG	0.167	0.121
Insulin	0.366^**^	< 0.0001
HOMA- IR	0.381^**^	< 0.0001
TG	0.117	0.277
TC	0.002	0.985
LDL-C	− 0.031	0.773
HDL-C	0.187	0.081
Adiponectin	0.231^*^	0.031
TNF-α	0.171	0.111
IL-6	0.268^*^	0.012

*<0.05, **<0.01

**Table 3:** The correlation analysis of various variables with CCN5 in the GDM group

Variables	*R*	*P*-value
Age	− 0.131	0.223
BMI	0.251^*^	0.019
SBP	0.030	0.782
DBP	0.059	0.585
FBG	0.273^**^	0.010
Insulin	0.369^**^	< 0.0001
HOMA-IR	0.424^**^	< 0.0001
TG	0.108	0.317
TC	0.093	0.391
LDL-C	0.107	0.323
HDL-C	0.022	0.839
Adiponectin	− 0.158	0.142
TNF-α	0.253^*^	0.018
IL-6	0.340^**^	0.001

### Association of CCN5 with risk of GDM and receiver operating characteristic analysis

Table 4 displays the outcomes of the binary logistic regression analysis, which show that in both the crude and adjusted models controlling for age and BMI, the risk of GDM significantly increased with every 10-unit change in CCN5 serum levels (OR = 1.273 [%95 CI (1.1890–1.364)] and OR = 1.292 [%95 CI (1.202–1.388)], respectively, *P* < 0.0001).

**Table 4:** Odd ratio of the GDM status based on 10-unit change in CCN5

Model	*B*	S.E	Wald	*P*	Odd ratio (*B*)	95% CI for odd ratio (*B*)	
						Lower	Upper
Crude	0.242	0.035	47.485	< 0.0001	1.273	1.189	1.364
Adjusted^a^	0.256	0.037	48.296	< 0.0001	1.292	1.202	1.388

^a^Adjusted for BMI and age

**Fig. 2:**
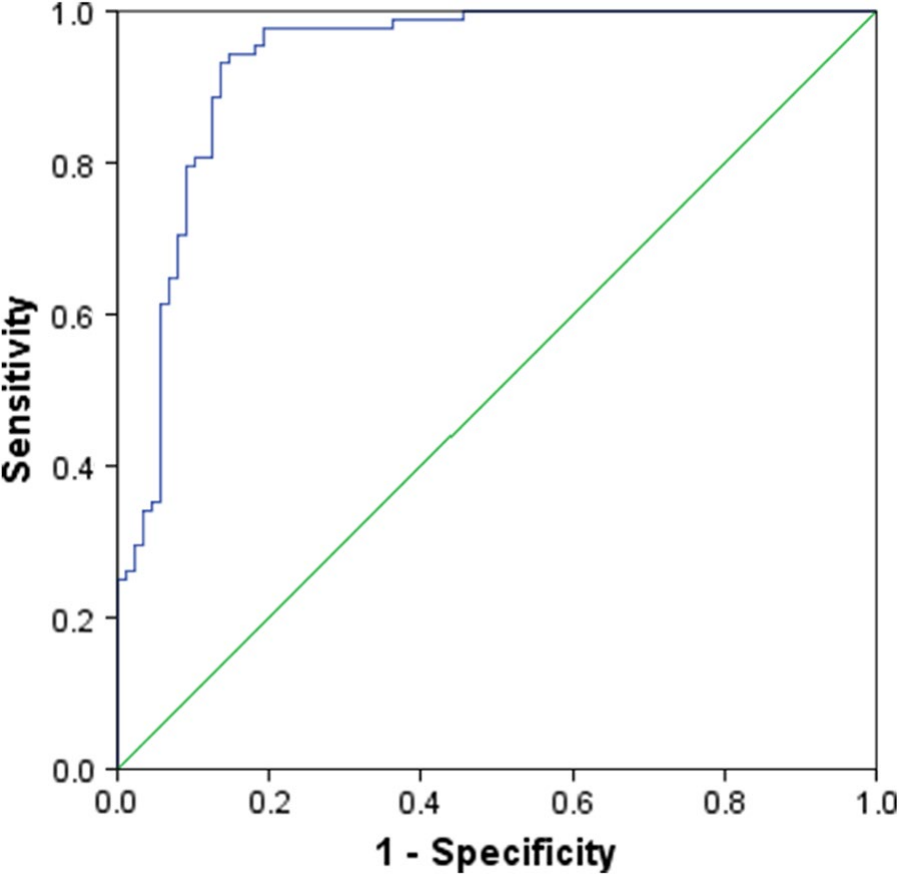
The analysis of ROC curve for CCN5 in the control and GDM groups. The best cut-off for CCN5 was 270.14 ng/ml for distinguishing GDM group from the control group (sensitivity and specificity were 94.3% and 85.2%, respectively). The area under the curve was estimated to be 0.932 [95% Cl (0.893–0.971)], (*P* < 0.0001)

Figure 2 illustrates the ROC curve analysis of CCN5 in both the control and GDM groups. The optimal cut-off for CCN5 was determined to be 270.14 ng/mL to differentiate the group with GDM from the control group, with a high sensitivity of 94.3% and specificity of 85.2%. Additionally, the area under the curve was estimated to be 0.932 [95% CI (0.893–0.971)], indicating a high level of accuracy (*P* < 0.0001).

## Discussion

GDM is a common complication during pregnancy, with obesity and insulin resistance being the major contributing factors [2, 8]. The development of GDM may involve the release of cytokines and chemokines from adipose tissue [14]. Prior research has explored the plausible involvement of CCN family constituents in the pathogenesis of metabolic disorders, including but not limited to obesity and diabetes. [24], and some studies have also found an association between CCN3 and WISP1/CCN4 serum levels with GDM [25, 26]. Given the known relationship between CCN5 and obesity and insulin resistance, we hypothesized that CCN5 serum levels may be associated with the risk of developing GDM. Thus, this study aimed to compare CCN5 serum levels in pregnant females without and with GDM and to investigate its correlation with inflammatory cytokines.

Our investigation revealed a notable association between the serum concentration of CCN5 and the susceptibility to GDM. Specifically, GDM patients exhibited an elevated level of CCN5 in their serum, in contrast to the control cohort. This suggests that CCN5 may play a role in the development of GDM, as there was a direct and significant correlation between CCN5 and several risk factors for GDM, including BMI, insulin resistance index, FBG, and inflammatory cytokines (IL-6 and TNF-α). To the best of our understanding, the potential connection between serum levels of CCN5 and the susceptibility to GDM has yet to be examined. Although various studies have delved into the link between CCN5 and metabolic disorders, insulin resistance, and hypertrophic obesity, such an investigation remains unexplored. Prior studies have established a positive correlation between the expression of CCN5 and ectopic fat accumulation, while conversely noting a negative correlation with insulin sensitivity [19]. Furthermore, it was observed that the transcription of the CCN5 gene exhibited heightened activity within the subcutaneous adipose tissue of individuals affected by metabolic disorders [27].

Our research provides supporting evidence for prior investigations by indicating a favorable association between CCN5 concentration and BMI among individuals with GDM. At the molecular level, the mechanism of CCN5's dual action in adipogenesis has been elucidated. Increased expression and secretion of CCN5 in adipose progenitor cells lead to elevated WNT signaling in subcutaneous abdominal adipose tissue, which in hypertrophic obesity with insulin resistance and accumulation of visceral fat may lead to the suppression of peroxisome proliferator-activated receptor gamma (PPARγ) [19, 28]. PPARγ is critical for adipogenesis and differentiation of adipocytes. When its expression is inhibited, adipogenesis stops, and adipocytes hypertrophy, increasing the risk of cardiovascular diseases, diabetes, and insulin resistance [29]. Previous findings have also reported a reduction in PPARγ expression under the influence of TNF-α [30].

The mechanisms described provide a justification for the observed direct and significant correlation between CCN5 and HOMA-IR and TNF-α in our study. In another pathway, CCN5 gene knockout resulted in the commitment of NIH 3T3 fibroblasts to the adipose lineage and the differentiation of human preadipocytes. In this pathway, bone morphogenetic protein 4 (BMP4) facilitated the nuclear entry of ZNF423 by dissociating WISP2 from the Wisp2/Zfp423 complex, which in turn participate in the activation of PPARγ and differentiation of cells toward the adipose lineage [28, 31]. Therefore, it is plausible that increased expression of CCN5 could inhibit PPARγ and subsequently contribute to insulin resistance and diabetes through the formation of an additional complex with Zfp423. In contrast, CCN5 has been shown to improve metabolic status by acting on pancreatic beta cells. In vitro studies have elucidated the mechanism by which CCN5 improves pancreatic cell function. Adipose tissue serves as a reservoir for a variety of growth factors, amongst which is insulin-like growth factor-1 (IGF-1) [32, 33], and its expression is elevated in GDM patients [34]. In a mouse pancreatic islet study, gene expression and protein levels of CCN5 were upregulated under the influence of IGF-1, leading to the activation of AKT and ERK2 and contributing to the growth and survival of pancreatic beta cells, thus improving the condition of diabetes [17, 35]. Additionally, CCN5 gene knockout-induced mild obesity and diabetes were observed in another study, with increased FBG levels in CCN5 knockout mice [21]. In our investigation, we have detected a notable association between CCN5 and FBG levels in the serum, which stands in opposition to prior scholarly findings indicating that CCN5 could impede the onset of obesity and diabetes by repressing the TGF-β signaling cascade [21]. TGF-β has been shown to activate the downstream gene Smad3, which inhibits the expression of PPARγ coactivator-1α (PGC-1α) gene, potentially leading to mild obesity in individuals with reduced CCN5 expression [22]. However, Grünberg et al*.* [18] observed improved metabolic markers in aP2-CCN5 transgenic mice, including a decrease in FBG levels and increased insulin sensitivity when fed a high-fat diet. These improvements were attributed to the activation of GLUT4 and ChREBP, leading to elevated levels of metabolically useful fatty acid esters such as FAHFAs and PAHSAs. The upsurge in lipogenesis indicators is deemed to have played a role in the amelioration of insulin sensitivity and the decrement of fasting blood glucose levels that were observed in the genetically modified mice [18].

To further understand the role of CCN5 in GDM, more investigation is warranted to determine if the increase in CCN5 serum levels is a compensatory mechanism to improve the disease or if CCN5 plays a role in its pathogenesis. Future studies should investigate the intricate mechanisms that underlie the pathogenesis of GDM; moreover, a comprehensive analysis of the impact of CCN5 on pancreatic cells and its influence on insulin secretion is warranted. Equally important is to examine the potential interplay between CCN5, the receptor and signaling pathways of insulin, and the adipose, liver, and muscle tissues. These investigations can provide new perspectives on the role of CCN5 in GDM. However, due to limitations in the present study, the pathways related to CCN5 were not investigated.

## Conclusion

In the present investigation, our aim was to explore the potential correlation existing between the concentrations of CCN5/WISP2 in the serum and the likelihood of developing GDM, which had not been previously explored. Our findings indicate that CCN5 levels were significantly higher in women with GDM compared to pregnant females without GDM. It remains unclear whether this increase is a compensatory mechanism for managing GDM complications or directly involved in the pathogenesis of the disease. Based on our findings, there appears to be a noteworthy and affirmative correlation between serum levels of CCN5 and the risk of developing GDM, as well as its associated risk factors such as BMI, insulin resistance index, FBG, and inflammatory cytokines TNF-α and IL-6. Perhaps, the link between CCN5 and GDM is related to the positive correlation of CCN5 with inflammatory cytokines and insulin resistance as probable mechanisms. These results indicate that CCN5 may potentially contribute to the pathophysiology of GDM. Further investigations are needed to gain a deeper understanding of this association.

## Data Availability

The datasets used and/or analyzed during the study available from the corresponding author on reasonable request.
